# Neuromuscular Blocking Agents in Anesthesia: A Narrative Review of Contemporary Challenges and Reversal Approaches

**DOI:** 10.3390/jcm15093513

**Published:** 2026-05-04

**Authors:** Paweł Radkowski, Marta Jutrzenka, Maciej Szewczyk, Alicja Witkowska, Marcin Muża, Dariusz Onichimowski, Łukasz Grabarczyk

**Affiliations:** 1 Department of Anesthesiology and Intensive Care, Faculty of Medicine, Collegium Medicum, University of Warmia and Mazury in Olsztyn, 10-561 Olsztyn, Poland; pawel.radkowski@uwm.edu.pl (P.R.); onichimowskid@wp.pl (D.O.); 2 Department of Anesthesiology and Intensive Care, Regional Specialist Hospital in Olsztyn, 10-561 Olsztyn, Poland; 3 Department of Anesthesiology and Intensive Care, Hospital Zum Heiligen Geist in Fritzlar, 34560 Fritzlar, Germany; 4 Department of Medicine, Collegium Medicum, University of Warmia and Mazury in Olsztyn, 10-561 Olsztyn, Poland; jutrzenkamarta@gmail.com; 5 Department of Internal Medicine, Independent Public Multispecialty Health Care Center, 73-110 Stargard, Poland; 6 Department of Medicine, Medical University of Łódź, 90-419 Łódź, Poland; alicja.witkowska1@stud.umed.lodz.pl; 7 Faculty of Health Sciences, Powiślańska Academy of Apllied Sciences, 82-500 Kwidzyn, Poland; m.muza1@powislanska.edu.pl; 8 Department of Anesthesiology and Intensive Care, Ceynowa Specialist Hospital, 84-200 Wejherowo, Poland; 9 Alarm Clock Clinic, Coma Recovery and Neurorehabilitation Center, Kondratowicza 8, 03-242 Warszawa, Poland

**Keywords:** neuromuscular depolarizing agents, sugammadex, neostigmine, medical errors, anesthesiology

## Abstract

Medical errors are inevitable and will happen to almost every specialist. In anesthesiology, one of the main concerns is the inappropriate application of muscle relaxants (MRs). As this group of drugs plays a significant role in facilitating endotracheal intubation and optimizing surgical conditions, it is widely and commonly used in the medical field. To prevent residual neuromuscular block, anesthesiologists may pharmacologically reverse the neuromuscular block (NMB) by administering reversal agents. Lately, sugammadex is becoming more popular due to its ability to reverse various levels of NMB more rapidly than traditionally used acetylcholinesterase inhibitors such as neostigmine. The common challenges and errors associated with the administration of neuromuscular blocking agents (NMBAs) and muscle reversal agents include the absence of neuromuscular monitoring, underestimation of the residual block (RB), misinterpretation of DUR25, inappropriate size descriptors for muscle relaxants and reversal agents requiring weight-based dosing, the wrong dosing of rocuronium, poor usage of cisatracurium among patients with renal or hepatic failure, and the wrong usage of succinylcholine. Another source of mistakes may be inaccurate knowledge about the pharmacokinetics and pharmacodynamics of the administered drugs. Medication errors may occur not only when it comes to the usage of muscle relaxants but also with the use of reversal agents, including lack of neuromuscular monitoring, choosing the wrong antagonist strategy, “too early” administration of neostigmine, inappropriate dosing, and insufficient knowledge about drug interactions. Improving the knowledge of administered drugs and adhering to the latest recommendations could prevent many complications. This article aims to review the current challenges in the use of muscle relaxants and reversal agents in anesthesia.

## 1. Introduction

The administration of neuromuscular blocking agents (NMBAs) is essential to achieve adequate muscle relaxation during surgical procedures [1]. Different surgeries require varying degrees of neuromuscular blockade. Muscle relaxants are commonly used to aid in performing specific types of surgical procedures, such as abdominal surgery. NMBAs are classified as depolarizing or non-depolarizing, with the latter further divided into steroidal, benzylisoquinolinium, and mixed-onium chlorofumarate compounds [1,2]. Understanding their pharmacodynamics and pharmacokinetics enables clinicians to select the most appropriate agent for each patient. Reversal agents are crucial for safely terminating neuromuscular blockade.

### Background

Medical errors, a leading cause of death in the U.S., can harm patients and negatively affect healthcare providers’ mental health, leading to anxiety, fear, or depression [3]. Anesthesiology is particularly vulnerable to medication errors, including incorrect NMBA administration. A survey of 295 anesthesiologists in Australia and New Zealand found that over 95% reported experiencing drug errors, with 25.6% involving muscle relaxants [1,4,5]. Despite safety measures such as syringe coding, double-checking, and legible labeling, errors continue to occur and require ongoing attention [4]. It is important to note, however, that this survey is subject to several potential biases, including sampling/nonresponse bias (only 30% responded, and non-respondents may differ systematically), recall bias (errors may be forgotten or selectively reported), selection bias (findings reflect only those who chose to respond), and lack of denominators/contextual bias (no data on total cases, emergency cases, or workload, limiting interpretation of incidence and contributing factors) [4]. Anesthesiologists face numerous challenges in performing and managing neuromuscular blockade, including ensuring drug safety, optimizing dosing and monitoring, preventing residual paralysis, and minimizing medication errors.

## 2. Materials and Methodology

This narrative review was conducted to summarize current knowledge on neuromuscular blocking agents (NMBAs), their monitoring, and reversal strategies in anesthesia and intensive care. A structured literature search was performed in PubMed, Google Scholar, the Cochrane Library, and institutional research platforms. Keywords included “muscle relaxants,” “neuromuscular blockade,” “depolarizing/non-depolarizing NMBAs,” “reversal agents,” “succinylcholine,” “rocuronium,” “atracurium,” “neostigmine,” “sugammadex,” “train-of-four,” “TOF,” and “intensive care unit,” alone or in combination. Only articles in English or with English abstracts were considered. Drug monographs and peer-reviewed pharmacological sources were also consulted. Both recent studies and foundational literature were included to provide a comprehensive overview of NMBA use, monitoring, and reversal in contemporary clinical practice.

The aim of this paper is to present the common challenges in anesthesia during the usage of muscle relaxants and reversal agents.

## 3. Pharmacological Overview

**Figure 1:**
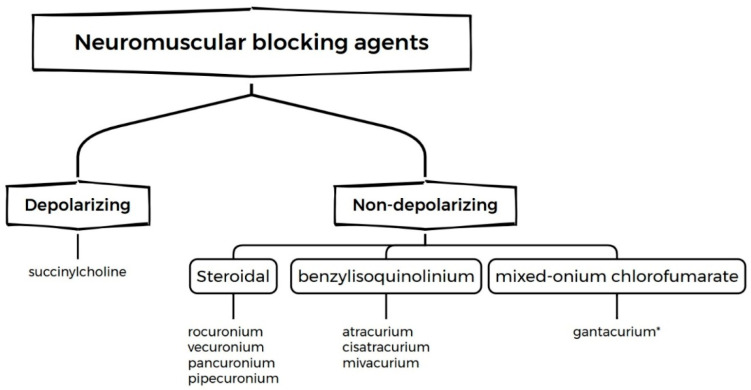
Schematic classification of neuromuscular blocking agents by chemical structure and mechanism of action. * unregistered drug.

Muscle relaxants are primarily used in anesthesiology to induce neuromuscular block for intubation. Despite their routine utilization, their pharmacokinetics and pharmacodynamics remain complex, presenting challenges for monitoring, safe use, and reversal. Muscle relaxants are subdivided into depolarizing and non-depolarizing agents (Figure 1) based on their mechanism and duration of action at the neuromuscular junction [6].

Depolarizing agents act as acetylcholine receptor agonists [7], causing sustained depolarization of the neuromuscular endplate and neuromuscular blockade. The traditional distinction between a pure phase I (depolarizing) and phase II (desensitizing) block is an oversimplification as evidence suggests that succinylcholine-induced neuromuscular block represents a continuum of effects rather than two clearly separable phases [8]. In a letter to the editor, Lu et al. [9] in 2004 stated that so-called phase II block, in which the characteristics of neuromuscular blockade resemble those of nondepolarizing agents and the duration of blockade is prolonged, may occur following the administration of high doses of succinylcholine (e.g., 2–5 mg/kg). However, as an answer, Naguib [10] wrote that phase II block is not a dose-related phenomenon.

Depolarizing agents are resistant to acetylcholinesterases and primarily metabolized by plasma cholinesterase to succinate and choline. Succinylcholine is a representative depolarizing agent and characterized by rapid onset (30–60 s) and a short duration of action (5–10 min), as summarized in Table 1 [11,12,13,14,15]. Nevertheless, its use is associated with several potential complications, including hyperkalemia, bradyarrhythmias, increased intraocular pressure, postoperative myalgia, and malignant hyperthermia in susceptible individuals. Furthermore, patients with cholinesterase deficiency may experience significantly prolonged paralysis, posing an anesthetic safety concern [11,12,13].

Non-depolarizing agents are competitive antagonists that bind to the alpha subunits of nicotinic receptors on the postsynaptic membrane, thereby blocking acetylcholine (Ach) binding. These agents are further classified into steroidal components, such as rocuronium, vecuronium, and pancuronium, as well as benzylisoquinolinium derivatives, including cisatracurium, atracurium, and mivacurium. They also significantly differ in onset, duration, and metabolism, as summarized in Table 1 [2,11,12,13,14,15,16,17,18,19,20,21,22].

The most commonly used reversal agents for non-depolarizing neuromuscular blocking agents include acetylcholinesterase inhibitors such as neostigmine, edrophonium, and pyridostigmine. These agents act by inhibiting the breakdown of acetylcholine at the neuromuscular junction, thereby increasing its concentration and competitively displacing the neuromuscular blocking agent from the nicotinic receptor. However, acetylcholinesterase inhibitors also increase muscarinic activity, always necessitating co-administration of anticholinergic drugs like atropine or glycopyrrolate to counteract side effects such as bradycardia or increased secretions.

More recently, sugammadex, a modified γ-cyclodextrin, has revolutionized reversal strategies, particularly for rocuronium and vecuronium [23]. Sugammadex (SM) encapsulates free drug molecules in the plasma, effectively reducing their concentration and allowing neuromuscular transmission to resume. It provides a faster and more predictable reversal, even of profound blocks, with a lower risk of muscarinic side effects.

Neostigmine is the only anticholinesterase in routine use. It requires the simultaneous use of an anticholinergic agent, such as glycopyrrolate or atropine, to prevent its muscarinic effects. Neostigmine has a ceiling effect: increasing its dose does not necessarily increase its efficacy, which is a limitation of its use [24].

Cysteine, a naturally occurring amino acid, is under investigation as a specific reversal agent for gantacurium, an ultra-short-acting non-depolarizing agent not yet widely available [25]. Cysteine works by forming an inactive adduct with gantacurium, allowing for rapid inactivation [26].

While the pharmacology of muscle relaxants is well understood in theory, their clinical use still presents several challenges. Pharmacokinetic challenges might include organ dysfunction, Hofmann elimination variability, altered volume of distribution, as well as genetic variants.

Butyrylcholinesterase (BChE) deficiency is inherited in an autosomal recessive manner, and numerous variants of the *BChE* gene have been identified, many of which lead to the reduced ability to metabolize choline esters. Approximately 25% of Caucasians carry mutations in the *BChE* gene. It is estimated that about 76% of the population is homozygous for the wild-type *BChE* allele, whereas 24% carry at least one variant allele. Most of these genetic variants are associated with decreased enzyme activity. These polymorphisms can significantly influence the metabolism of certain muscle relaxants, particularly succinylcholine and mivacurium, both of which are hydrolyzed by BChE. As a result, individuals carrying variant alleles may experience prolonged neuromuscular blockade and an increased risk of related adverse effects [27,28,29]. Available evidence suggests that genetic polymorphisms can influence the pharmacokinetics and pharmacodynamics of rocuronium. Variants in genes encoding anion-transporting polypeptides, such as SLCO1B1 and SLCO1A2, have been associated with prolonged duration of neuromuscular block and delayed recovery. By contrast, patients carrying the ABCB1 rs1128503 CT and CC genotypes exhibit a shorter duration of rocuronium action and faster recovery compared with those with the TT genotype. Moreover, an extended clinical duration of rocuronium has been observed in individuals with NR1I2 rs2472677 and rs6785049 polymorphisms [29].

Acquired pseudocholinesterase deficiency may develop in various clinical conditions and with exposure to certain drugs. Reduced enzyme activity or production can be seen after usage of corticosteroids, monoamine oxidase inhibitors, anticholinesterase agents, and various chemicals, including organophosphate insecticides. It can be also seen in malnutrition, pregnancy and the postpartum period, burns, liver and kidney disease, hemodialysis, myocardial infarction, congestive heart failure, malignancy, and chronic infections [30].

Pharmacodynamic alterations might instigate inconsistent sensitivity, residual neuromuscular blockade, the ceiling effect of reversal agents, as well as drug interactions. These factors highlight the need for individualized dosing strategies, close neuromuscular monitoring, and appropriate agent selection, especially in high-risk cases.

## 4. Monitoring and Residual Blockade Challenges

One of the sources of errors during the administration of NMBAs is the absence of neuromuscular monitoring or/and incorrect interpretation of the results. Muscle relaxants present a wide variety of durations of action, depending on the manufacturer and the patient’s overall state, which should be objectively monitored [31]. Neuromuscular monitoring is recommended and should be performed whenever NMBAs are used. Neuromuscular monitoring devices should be placed at each site providing anesthesia and post-anesthesia care unit [32].

Quantitative monitors objectively measure the train-of-four ratio (TOFR) in real time, while qualitative monitoring with the usage of a peripheral nerve stimulator (PNS) is based on an anesthesiologist’s subjective evaluation of muscle contractions only by visual or tactile observation in response to TOF stimulation [33,34]. Objective monitoring is preferred and recommended, but a study from 2013 revealed that only 17% of anesthetic practitioners routinely use it [35]. At this point, it also should be pointed out that the terms nerve simulator and monitor, should not be used interchangeably [36].

In addition, it should be remembered that clinical tests (5 s head lift, gagging on the tracheal tube, presence of normal tidal volume, response to verbal command, etc.) and subjective monitoring do not guarantee reliable monitoring of neuromuscular recovery [33,37]. They are valuable; however, they cannot replace objective monitoring methods. More than 70% of patients with TOFR < 0.7 will show an ability to sustain the head for 5 s [33]. Also, some patients may reach the normal tidal volume and manifest spontaneous breathing when the TOFR is as low as 0.2 [38].

Using neuromuscular monitoring is beneficial during various phases of the perioperative period, and the gold standard is the evaluation of the adductor pollicis response to train-of-four (TOF) stimulation at the ulnar nerve because, among all sites, it most accurately reflects pharyngeal muscle recovery [32,34]. During some procedures, the corrugator supercilii muscle is a more available site, but its usage is associated with a higher risk of the postoperative residual neuromuscular blockade—it recovers earlier than the diaphragm and upper airway muscles, which creates a clinically important mismatch during recovery [33]. At the induction of anesthesia, loss of TOF response (TOF = 0) signals the appropriate intubation time to reduce the possibility of vocal cord damage [30]. Intraoperative neuromuscular monitoring allows achieving optimal surgical conditions, enables appropriate NMBA dosing, and is a reliable tool for choosing NMBA reversal agents [33,39]. Neuromuscular monitoring is especially useful for determining whether the recovery from neuromuscular blockade is sufficient to perform extubation (TOFR ≥ 0.9), and it decreases the possibility of residual block and its complications [33,37,39].

Residual neuromuscular blockade (RNMB) refers to the continued presence of neuromuscular weakness following the use of muscle relaxants, extending into the perioperative period and beyond the operating room. It is typically identified through objective monitoring. Assessment of clinical signs is useful and widely applied in daily practice; however, it should be noted that it is a subjective method for evaluating residual neuromuscular blockade. Only objective monitoring allows reliable identification of RNMB [40]. RNMB is still considered a significant but frequently overlooked or underestimated clinical problem, associated with serious and life-threatening complications such as airway obstruction, the need for reintubation, hypoxia, or unpleasant symptoms of muscle weakness [36,39]. Despite routine administration of anticholinesterase reversal agents, up to 40% of patients arrive in the post-anesthesia care unit with residual curarization [36]. RNMB also occurs in the pediatric population, where the incidence of residual paralysis is around 28% (based on the study from 2015) [41]. RNMB increases postoperative mortality, morbidity, length of hospitalization, and cost of treatment [32,38]. Based on guidelines, only quantitative monitoring of the adductor pollicis can be used to assess RNMB. A ratio between the fourth and first TOF response (T4/T1) of ≥ 0.9 is needed to exclude the diagnosis of RNMB [32].

RNMB is significant in all age groups. Pietraszewski et al. [42] conducted a study with 415 elderly patients. TOF ratios below 0.7 were observed in 31% of all patients upon arrival to the post-anesthesia care unit, despite apparent full clinical recovery. Notably, PORC was significantly more frequent in elderly patients (44%) compared to their younger counterparts (20%) (*p* < 0.05). This finding is clinically relevant, as elderly individuals demonstrate a higher incidence of hypoxia in the PACU (17.9% vs. 8.2%), indicating impaired respiratory function due to incomplete neuromuscular recovery [42].

Additionally, peripheral muscles have different sensitivities to NMBAs than respiratory muscles, which is associated with various onsets, times of duration, and recovery times [39,43,44]. Studies investigating the onset of neuromuscular blockade across different muscles show marked heterogeneity, as they employ various agents and doses, differ in methodology, and often compare distinct muscle groups. Consequently, it is challenging to determine exact time intervals for the development of complete neuromuscular block. For instance, Plaud et al. [45] examined neuromuscular block at the laryngeal adductor muscles and the adductor pollicis in 22 adult patients with anesthesia induced and maintained using propofol and alfentanil. Following administration of 0.07 mg/kg mivacurium, the mean onset time was 151 ± 40 s at the larynx and 241 ± 79 s at the adductor pollicis. When the dose was increased to 0.14 mg/kg, onset remained faster at the vocal cords (137 ± 20 s) than at the adductor pollicis (201 ± 59 s, *p* < 0.01) [45].

In another investigation, Hemmerling et al. [46] simultaneously measured neuromuscular block after 0.2 mg/kg mivacurium at several sites—the larynx, diaphragm, adductor pollicis, orbicularis oculi, and corrugator supercilii. They found that the respiratory muscles (larynx and diaphragm) reached a mean maximum block of 99% within 80–90 s, whereas the orbicularis oculi and corrugator supercilii required more than 150 s, and the adductor pollicis required over 3 min. Based on their findings, the order of increasing onset time was as follows: diaphragm → larynx → corrugator supercilii → orbicularis oculi → adductor pollicis [46]. The order of muscles affected after the administration of non-depolarizing NMBAs is shown in Table 2 [43,44]. The muscles recover from neuromuscular blockade to normal function in the same order, so respiratory movements can be observed, while pharyngeal muscles are still paralyzed, so the presence of spontaneous breathing is not a reliable sign to perform extubation [38].

## 5. Challenges in Using Size Descriptors for Weight-Based Dosing of Muscle Relaxants

Administering the appropriate dose of muscle relaxants requiring weight-based dosing may be challenging due to the lack of clear recommendations. Also, patients with obesity, which includes up to 20% of ICU patients, should have different size descriptors, but no size descriptor is undoubtedly better than others and should be used as a universal one [47,48]. In comparison to the ideal body weight (IBW), which estimates a fat-free mass using only height, and lean body weight (LBW), which is calculated based on an individual’s height and weight, the adjusted body weight (ABM) attempts to take into consideration the additional lean weight in obese patients [48]. The use of total body weight in obese patients may be a cause of drug overdose and may significantly prolong the duration of neuromuscular blockade [49,50]. While for non-depolarizing NMBAs, it is suggested to use IBW, ABW, or LBW, and the dosing of succinylcholine should be based on actual body weight [48,51]. The suggested size descriptors for dosing of muscle relaxants and reversal agents are shown in Table 3 [32,48,51].

## 6. Knowledge of Pharmacokinetics and Pharmacodynamics of Administered Muscle Relaxants

It is crucial for anesthesiology practitioners to know the pharmacokinetics and pharmacodynamics of the administered drugs. Despite the well-known fact that neuromuscular blockade agents do not have sedative or amnestic properties, some reports in the literature suggest that more than 30% of patients in intensive care units experienced unintended awareness after the administration of NMBAs [36]. However, a meta-analysis published in 2021 on awareness with paralysis in mechanically ventilated patients in the emergency department and intensive care unit estimated the incidence of unintended awareness to be 12.3% (2.8–26.0%, 95% CI). The authors noted, however, that the included studies varied considerably in methodology and quality. When analyzing subgroups considered to be of higher quality, the estimated incidence of awareness was 1.9–3.4%. Nevertheless, this remains a highly important issue, as retained consciousness in a paralyzed, mechanically ventilated patient is a profoundly traumatic experience. Therefore, special attention should be paid to this aspect when performing neuromuscular blockade [52].

Also, the duration of action of NMBAs varies among patients. Both age and comorbidities may have significant impacts on this process. However, it should be emphasized that these are only some of the factors influencing the course of neuromuscular blockade. They are not the only determinants, and due to the multitude of influencing variables, it is not possible to precisely predict how neuromuscular blockade will evolve. Therefore, the use of objective neuromuscular monitoring is essential.

Studies have shown gender differences in the pharmacokinetics of NMBAs. For example, females show around 20–30% greater sensitivity to the effects of vecuronium, rocuronium, and pancuronium, so dose reduction is necessary whenever a short duration is the main goal [53,54].

In a review article on the effects of acid–base disturbances on the action of skeletal muscle relaxants, it was noted that electrolyte abnormalities such as hypokalemia, hypocalcemia, hypophosphatemia, and hypermagnesemia, as well as conditions including hypercalcemia, hyperkalemia, and alkalosis, are believed to reduce the intensity of neuromuscular blockade [2].

Conditions such as hypothermia and hypermagnesemia and use of inhalational anesthetics (apart from nitrous oxide) may change the duration of action of NMBAs and prolong it, while antibiotics, diuretics, antiarrhythmics, and electrolyte imbalances have a minor effect [38].

Additionally, NMBA usage with lithium might prolong and enhance the effect of neuromuscular blockade, including both depolarizing and non-depolarizing agents. Due to its chemical similarity to sodium (Na^+^), potassium (K^+^), magnesium (Mg^2+^), and calcium (Ca^2+^) ions, lithium can interfere with the distribution and kinetics of these electrolytes. It enters cells via sodium channels and tends to accumulate intracellularly. As Kishimoto et al. [55] reported, the usage of 50 mg of rocuronium in a patient taking 600 mg/day of lithium carbonate for bipolar disorder resulted in the TOF ratio decreasing to 0%. Administration of sugammadex was required, after which the TOF ratio improved to 95% [55].

In 2024, a meta-analysis was published on the use of intravenous calcium ions together with neostigmine for the reversal of neuromuscular blockade. It suggested that calcium may be co-administered with neostigmine during the early phase of neuromuscular blockade to facilitate and enhance the recovery of neuromuscular function. However, the authors emphasized that these findings require further confirmation, and additional studies are needed to clarify calcium concentrations as well as the dose–response relationships with neuromuscular blocking agents and acetylcholinesterase inhibitors [56].

In a 2025 review on the use of neuromuscular blocking agents in emergency departments, the use of magnesium sulfate as a pretreatment in RSI was partially evaluated. The authors indicated that available data suggest its administration may accelerate the onset of rocuronium action and the development of neuromuscular blockade. Consequently, intubation conditions achieved with this approach in RSI may be comparable to those observed with succinylcholine alone. It was also noted that magnesium sulfate may allow for a reduction in the dose of rocuronium while maintaining intubation conditions similar to those achieved with higher doses. At the same time, it was emphasized that comparable intubation conditions have also been reported in some studies without the use of pretreatment. The authors highlighted the need for further research to identify clinical situations in which patients may benefit from magnesium sulfate administration prior to rocuronium [57].

During the administration of mivacurium it should be remembered that this NMBA is characterized by a intermediate onset time of even up to 5 min [58].

## 7. Agent-Specific Challenges

Administration of the wrong dose of rocuronium may be the next cause of medical error. Due to the potentiating effect of volatile anesthetics on rocuronium, during procedures longer than an hour, some adjustments should be made, including using lower infusion rates or administering smaller maintenance doses at less frequent intervals. The recommendation for rocuronium dosing includes rapid sequence intubation; however, a study published in 2021 revealed that administering doses higher than or equal to 1.4 mg/kg was associated with a more effective first attempt, especially with the use of direct laryngoscopy and among patients with pre-intubation hypotension [59].

Interestingly, chronic statin therapy may also influence the pharmacodynamics of neuromuscular blocking agents. In a 2016 study by H. Ren et al. [60], the impact of statin use (for a minimum duration of 3 months) on rocuronium activity was investigated. The authors found that patients receiving statins experienced a significantly shorter onset time and a prolonged duration of neuromuscular blockade compared to the control group. These effects may be attributed to statin-induced alterations in muscle membrane properties or subclinical myopathy, which increase sensitivity to muscle relaxants [60].

Some anesthesiology practitioners use non-depolarizing NMBAs after the administration of succinylcholine, but such action may prolong the duration of blockage, while 1/3 of the intubation dose would be sufficient. However, when muscle relaxation caused by succinylcholine starts to decrease, non-depolarizing NMBAs should be administrated [41].

Due to the long list of the recognized side effects of succinylcholine and the highest risk of anaphylaxis among all NMBAs, some studies suggest reducing the use of this NMBA [58]. Some departments in Europe already report rare administration of this drug [59].

## 8. Priming, Timing, and Precurization

Furthermore, techniques such as priming (administration of a small dose of a neuromuscular blocking agent before the full dose), timing (administration of a neuromuscular blocking agent to an awake patient), and precurarization (administration of a small dose of a neuromuscular blocking agent prior to succinylcholine) are generally considered to have more disadvantages than benefits [57,58]. In priming and timing, patients may experience shortness of breath, and in the latter approach there may additionally be discomfort related to the effects of neuromuscular blockade while still conscious [50]. In precurarization, the prior administration of a neuromuscular blocking agent may attenuate the effect of succinylcholine and prolong its onset of action [57,58].

## 9. Insufficient Usage of Cisatracurium Among Patients with Renal/Hepatic Failure

In comparison to other steroid-based neuromuscular blocking agents, the predominant (77%) elimination mechanism of cisatracurium is Hoffman elimination, an organ-independent degradation that occurs in plasma and tissues [61,62]. It is recommended to consider this NMBA whenever the patient is affected by renal or hepatic failure because cisatracurium pharmacodynamics and pharmacokinetics are unaffected by the dysfunction of these organs, yet somehow the use of this muscle relaxant among these patients is insufficient [32,61,62].

## 10. The Most Common Challenges During the Usage of Reversal Agents

The use of reversal agents may be challenging, so the administration of these drugs should be guided by neuromuscular monitoring, which detects changes in neuromuscular blockade [13,34]. Also, it may be a valuable tool for not only choosing the antagonist strategy but also for determining the appropriate dose [34,39].

Neostigmine (NS) is considered a safe reversal agent, while in reality it is often administered too early in the absence of partial spontaneous neuromuscular recovery and may even prolong blockade [38]. It is recommended to obtain four responses to TOF (or TOF ratio ≥ 0.2) before administrating NS at a dose between 40 and 50 μg/kg adapted to ideal body weight [32]. Exceeding that dose may cause a ceiling effect [6]. One of the most noticeable downsides of neostigmine that should be remembered is its inability to reverse a deep and profound blockade (in any dose) [36,63,64]. To prevent potential side effects, neostigmine should be administrated with atropine or glycopyrrolate [64].

Sugammadex has been in use since 2008 and is commonly used to reverse the effect of steroidal NMBAs such as rocuronium and vecuronium. The advantage sugammadex over neostigmine shows in its lack of cholinergic effect and effective antagonization of any degree of blockade [65,66,67]. Additionally, a study from 2022 revealed that recovery in morbidly obese patients was faster after SM than after neostigmine, while the side effects associated with the usage of both agents were not significantly different [68]. The dosing of NMBAs in obese patients should be based on ideal body weight, but at the same time, some studies show that the dose should be around 40% higher than the dose counted for ideal body weight [32].

Anesthesiologists should be familiar with the possibility of sugammadex-induced anaphylaxis, which manifests within 5 min, most commonly with cardiovascular symptoms such as hypotension and tachycardia [69]. Based on guidelines, epinephrine should be the first-line treatment for perioperative anaphylaxis [65]. Severe and unexpected life-threatening adverse reactions to sugammadex are rare.

In a systemic review published in 2022 based on the selected studies included in further analysis, 25 patients were identified as having experienced an anaphylactic reaction. The most commonly reported symptom was hypotension (92%), followed by various forms of erythema (76%), decreased oxygen saturation (44%), tachycardia (40%), swelling/edema (28%), and wheezing (28%). Less frequently, other respiratory abnormalities such as hypercapnia were observed in two cases. Additionally, two cases of bradycardia were reported, associated with ST-segment depression and cardiac arrhythmias. Overall, clinical presentation appears relatively consistent across case reports. However, the true incidence and underlying mechanism of these reactions (i.e., sugammadex itself versus the rocuronium–sugammadex complex) remain unclear [70].

It is worth noticing that although rare, anaphylaxis is more common with sugammadex than with neostigmine [24,71].

Nevertheless, clinicians should remain aware of the potential for bradycardia and even asystole following its administration, particularly in light of reported cases of cardiac arrest after intravenous use of the drug.

Importantly, authors describing sugammadex-associated cardiac rhythm disturbances recommend that the drug be administered only when clearly indicated, in the lowest effective dose, and injected slowly under continuous ECG monitoring to help minimize the risk of such complications. Intravenous atropine and other vasoactive agents should be immediately available whenever sugammadex is used. Actually, glycopyrrolate may be worthy or even preferred over atropine, as it leads to less change in heart rate and it does not cross the blood–brain barrier. From the study by Cozanttis et al. [72], it appears that glycopyrrolate provides protection against cardiac effects resulting from intermittent administration of suxamethonium, comparable to or greater than that of atropine. Equally crucial is the responsibility to promptly report all suspected adverse drug reactions to appropriate pharmacovigilance authorities. Only through systematic reporting can the true incidence and clinical significance of the cardiovascular effects associated with sugammadex be fully understood [73,74].

One of the underappreciated facts about SM is its potential to reduce the effectiveness of contraceptive pills by binding estrogen and progestins. In vitro investigations using isothermal microcalorimetry have revealed that sugammadex binds strongly to steroid hormones, particularly progesterone. Given that progesterone is the active ingredient in many hormonal contraceptives, this interaction could theoretically lead to temporary inactivation of the hormone, potentially resulting in contraceptive failure and increasing the risk of unintended pregnancy. Nevertheless, the actual incidence of such events remains unclear [75].

A 2023 single-center survey explored this issue through a two-phase study, assessing anesthesia professionals’ awareness of the potential interaction between sugammadex and hormonal contraceptives, as well as evaluating perioperative counseling practices at a large teaching hospital. Among 234 patients receiving sugammadex, 48 (28%) were women of reproductive age who qualified for counseling. Notably, none of these 48 patient records documented discussions regarding possible contraceptive failure or the provision of relevant information, indicating limited awareness and counseling practices [76].

It is important to note that high-quality clinical data regarding the effect of sugammadex on oral hormonal contraceptives are currently lacking. A 2024 review emphasized the absence of solid evidence in this area. Two large retrospective database studies reported only two cases of postoperative pregnancy in women using hormonal contraceptives who received sugammadex during anesthesia [77]. The label of SM suggests that women taking hormonal contraception should use another contraceptive method or be abstinent for 7 days after SM exposure. Future studies could involve prospective trials with serial progesterone measurements before and after sugammadex administration in women on hormonal contraceptives undergoing elective surgery, which would help clarify the potential risk of unintended pregnancy [77]. Another important aspect regarding the use of sugammadex is its administration during non-obstetric surgical procedures in pregnant women. Theoretically, placental transfer and passage across the blood–brain barrier are limited; however, animal studies have yielded concerning results. Clinical data, however, are limited to small studies or case reports/series and do not allow for a definitive assessment of the drug’s safety. It is worth emphasizing that sugammadex can be extremely useful and even essential in certain clinical scenarios, such as cesarean section complicated by a “cannot intubate, cannot ventilate” situation. Further research is needed to adequately evaluate its safety in this context [77].

In 2018, the Safety Committee of the Japanese Society of Anesthesiologists (JSA) raised awareness about the need for correct SM dosing based on the patient’s body weight and depth of neuromuscular blockade, as there were cases of recurrence of neuromuscular blockade (recurarization) after SM administration. Additionally, a lack of neuromuscular monitoring is associated with an increased risk of recurarization [78]. Table 4 presents the recommended dosing of SM based on the depth of neuromuscular monitoring [5,78,79,80]. It should be emphasized that the doses presented in the table apply specifically to the reversal of neuromuscular blockade induced by rocuronium. This dosing regimen may not be appropriate for the reversal of blockade caused by other aminosteroidal neuromuscular blocking agents due to differences in binding affinity [81]. Sugammadex has a lower affinity for vecuronium, and the use of similar doses may result in recurarization in the post-anesthesia care unit (PACU) [82]. Conversely, the affinity of sugammadex for pipecuronium is higher, suggesting that lower doses may be sufficient to achieve effective reversal [83].

The newest reveal study with SM has revealed that unrestricted availability and a safer profile are not only associated with its increasing usage but also with increased rocuronium administration. It also corresponds with decreasing cisatracurium, atracurium, and suxamethonium use [84].

It is worth emphasizing, however, that current guidelines do not demonstrate clear superiority of one reversal agent over another—whether sugammadex or neostigmine. Sugammadex is recommended over neostigmine for reversal at deep, moderate, and shallow levels of neuromuscular blockade induced by rocuronium or vecuronium in order to reduce the risk of residual neuromuscular blockade. According to the 2023 American Society of Anesthesiologists (ASA) guidelines, neostigmine remains a reasonable alternative in cases of minimal blockade, defined as a train-of-four (TOF) ratio in the range of 0.4 to less than 0.9 [80].

## 11. Ongoing Research on New Neuromuscular Blocking Agents and Reversal Agents

Recent advancements in neuromuscular blocking agents are very promising, including the new series of neuromuscular blocking agents called chlorofumarates [23,64].

Chlorofumarates are new compounds that show promising onset, duration, and rapid reversal of neuromuscular blockade after L-cysteine administration [23,64].

Gantracurium (GW280430A) was the first chlorofumarate to be discovered. It demonstrated a rapid onset of action, comparable to that of rocuronium, and an ultrashort duration, similar to succinylcholine; however, its clinical development was limited due to significant histamine release [85]. It should be noted that previous studies have reported that gantacurium can induce significant histamine release at doses four times the ED95 [86]. Additionally, a search of ClinicalTrials.gov identified only a single study investigating gantacurium, conducted in 2006 (The Efficacy and Safety of Gantacurium Chloride for Injection in Tracheal Intubation in Healthy Adult Patients Undergoing Surgery Under General Anesthesia, ClinicalTrials.gov ID: NCT00235976), the results of which are not publicly available [87].

CW 1759-50 (RP3000) is a shorter-acting chlorofumarate; in vivo, it exhibits an onset of action comparable to that of rocuronium. In vivo, it adducts with L-cysteine within approximately 2.3 min, with a comparable duration to that of succinylcholine, averaging around 7.4 (±1.9) minutes. The administration of L-cysteine has been shown to accelerate recovery following either a single dose or continuous infusion of CW 1759-50 [88].

CW002 (RP1000) exhibits an onset of action comparable to that of rocuronium—approximately 90 s when administered at 1.8 × ED95 (0.14 mg/kg). Its clinical duration resembles that of atracurium, averaging around 34 min. Notably, neuromuscular blockade induced by CW002 can be rapidly and effectively reversed with intravenous L-cysteine at a dose of 50 mg/kg, achieving recovery within one minute. By contrast, neostigmine is ineffective in reversing deep neuromuscular block caused by CW002 [23,89].

There are also promising findings in the use of calabadions as reversal agents for neuromuscular blockade caused by both steroidal and non-steroidal NMBAs [44]. Calabadion 1 is a methylene-bridged glycouril tetramer with o-xylylene caps and four sulfonate groups, forming a C-shaped structure that binds neuromuscular blocking agents. While it effectively reverses both rocuronium and cisatracurium in rats, its affinity for rocuronium is lower than that for sugammadex. By contrast, calabadion 2 shows a higher potency, reversing deep neuromuscular block from rocuronium, vecuronium, and cisatracurium rapidly and dose-dependently, outperforming sugammadex in preclinical models [90].

Further research needs to be performed to determine how these new discoveries will impact clinical practice [51].

## 12. Scientific Anesthesiology Societies and Their Recommendations

It is of great importance to highlight the recommendations issued by international and national scientific societies, such as the European Society of Anaesthesiology and Intensive Care (ESAIC), the American Society of Anesthesiologists (ASA), the Société Française d’Anesthésie et de Réanimation (SFAR), and the Society of Critical Care Medicine (SCCM). The most recent comprehensive guidelines were published in 2023 by the ESAIC (“Peri-operative management of neuromuscular blockade: A guideline from the European Society of Anaesthesiology and Intensive Care”), while updated recommendations from the SCCM published in 2026 addressed the use of neuromuscular blocking agents (NMBAs) in acute respiratory distress syndrome (ARDS) [32,80,91,92].

The latest recommendations from the ASA and ESAIC are summarized in Table 5 [80,91].

## 13. Conclusions and Future Directions

Neuromuscular blocking agents (NMBAs) and their reversal agents remain indispensable components of modern anesthesia; however, their use continues to be associated with significant risks, including medication errors, residual neuromuscular blockade (RNMB), inadequate reversal, and potentially life-threatening postoperative complications. This review highlights that many of these adverse events are preventable and are frequently linked to insufficient neuromuscular monitoring, inaccurate dosing, or inadequate understanding of NMBA pharmacology and reversal strategies.

One of the most important conclusions emerging from the current evidence is that objective quantitative neuromuscular monitoring should be considered a standard of care whenever NMBAs are administered. Clinical signs alone are insufficient to exclude residual paralysis, and failure to use quantitative monitoring contributes significantly to the persistent incidence of RNMB. Monitoring of the adductor pollicis muscle with assessment of the train-of-four ratio (TOFR) remains the gold standard for evaluating recovery, and extubation should only be considered when TOFR ≥ 0.9 has been achieved.

This review also emphasizes the importance of individualized NMBA selection and dosing. Patient-related factors such as age, obesity, organ dysfunction, electrolyte disturbances, genetic polymorphisms, and concomitant medications may substantially alter the pharmacokinetics and pharmacodynamics of muscle relaxants. Inappropriate dosing strategies—particularly in obese patients or in individuals with renal or hepatic dysfunction—may prolong neuromuscular blockade and increase the risk of postoperative complications. Cisatracurium, due to its organ-independent Hofmann elimination, remains particularly valuable in critically ill patients and in those with hepatic or renal impairment.

Among reversal strategies, sugammadex has significantly changed contemporary anesthetic practice by enabling rapid and reliable reversal of rocuronium- and vecuronium-induced blockade, including deep neuromuscular block. Nevertheless, its use requires awareness of potential complications such as recurarization due to underdosing, bradycardia, anaphylaxis, and possible interactions with hormonal contraception. Neostigmine remains an effective and appropriate option in selected patients with minimal residual blockade, but its efficacy is limited in deep neuromuscular block and inappropriate administration may paradoxically worsen recovery.

This review further demonstrates that safe neuromuscular management requires not only pharmacological knowledge but also strict adherence to evidence-based guidelines issued by international anesthesiology societies. Implementation of standardized monitoring protocols, optimization of dosing strategies, and continued education regarding NMBA pharmacology may substantially reduce preventable complications and improve perioperative patient safety.

Finally, ongoing research into novel neuromuscular blocking and reversal agents, including chlorofumarates and calabadions, may further improve the safety and precision of neuromuscular blockade management in the future. However, additional clinical studies are necessary before these agents can be incorporated into routine clinical practice.

## Figures and Tables

**Table 1:** Summary of depolarizing and non-depolarizing muscle relaxants covering their class, examples, mechanisms, onset, duration, metabolism, and clinical notes.

Drug	Class	Mechanism of Action	Onset Time	Duration	Metabolism & Exertion	Clinical Notes
Depolarizing						
Succinylcholine		Nicotinic ACh receptor agonist presenting with persistent depolarization of motor endplate, inactivation of Na^+^ channels, and flaccid paralysis	Rapid (0.5–2 min)	Short-acting (5–10)	Plasma butyrylcholinesterase; partly excreted in unchanged form in urine	Commonly used for rapid sequence intubation; minimal cardiovascular effects
Non-depolarizing						
Rocuronium	Aminosteroid	Competitive antagonist at nicotinic ACh receptor	Rapid onset (1–2 min) *	Intermediate (20–70 min)	Excreted by bile and kidneys	Commonly used for rapid sequence intubation; minimal cardiovascular effects
Vecuronium	Aminosteroid	Competitive antagonist at nicotinic ACh receptor	Intermediate (2–3 min)	Intermediate (20–60 min)	Excreted by bile and kidneys	Cardiovascularly stable; minimal histamine release
Pancuronium	Aminosteroid	Competitive antagonist at nicotinic ACh receptor	Slow (3–5 min)	Long (60–90 min)	Excreted by kidneys, partly by bile, partially metabolized by liver	Vagolytic effects, tachycardia; prolonged duration
Pipecuronium	Aminosteroid	Competitive antagonist at nicotinic ACh receptor	Slow (3–5 min)	Long (45–90 min)	Renal excretion	Minimal cardiovascular effects
Atracurium	Benzylisoquinolinium	Competitive antagonist at nicotinic ACh receptor	Intermediate (1–1.5 min) **	Intermediate (45–60 min)	Hofmann elimination, ester hydrolysis	Can cause histamine release, hypotension, flushing
Cisatracurium	Benzylisoquinolinium	Competitive antagonist at nicotinic ACh receptor	Intermediate (2–3 min)	Intermediate (60–90 min)	Hofmann elimination, ester hydrolysis	Preferred in renal/hepatic failure; very stable cardiovascular profile
Mivacurium	Benzylisoquinolinium	Competitive antagonist at nicotinic ACh receptor	Intermediate (2–3 min)	Short (25–45 min)	Rapid hydrolysis by plasma cholinesterase	Histamine release common; short duration
Doxacurium	Benzylisoquinolinium	Competitive antagonist at nicotinic ACh receptor	Slow (3–5 min)	Long (60–90 min)	Renal excretion	Rarely used; minimal cardiovascular effects
Gantacurium	Benzylisoquinolinium (experimental)	Competitive antagonist at nicotinic ACh receptor	Ultra-rapid (<1 min)	Ultra-short (5–10 min)	Rapid inactivation by chemical degradation	Promising rapid onset/reversal; “new agent”– few animal model studies conducted, probably human model studies ended in early stages

Notes, abbreviations: * dose-dependent onset of action; ** onset time only at higher doses (0.6–1 mg/kg).

**Table 2:** The order of muscles affected by non-depolarizing muscle relaxants.

Group of Muscles	Onset of the Block
Pharyngeal muscles	Slower
Masseter	
Genioglossus	
Adductor pollicis muscle	
Abdominal muscles	Moderate
Orbicularis oculi	Faster
Corrugator supercilii	
Diaphragm	

**Table 3:** Recommended size descriptors for muscle relaxants and reversal agents in obese patients.

Drug Name	Size Descriptor
Rocuronium	IBW [48]
Vecuronium	IBW [49]
Atracurium	IBW [48], LBW [49]
Succinylcholine	Actual body weight [49]
Sugammadex	IBW/IBW + 40% [28]

IBW—ideal body weight, LBW—lean body weight [32,48,51].

**Table 4:** Recommended doses of sugammadex for reversal of neuromuscular blockade based on neuromuscular monitoring [5,78,79,80].

Level of Neuromuscular Blockade	Neuromuscular Monitoring	Sugammadex Dose (mg/kg)
Shallow	TOF count = 4, TOF fade present	2
Moderate	TOF count = 2 or 3	2
Moderate	TOF count = 1	4
Deep	TOF count = 0, Post Tetanic Count ≥ 1	4

TOF—Train of Four.

**Table 5:** Summary of the 2023 recommendations from American and European anesthesiology societies regarding the use of skeletal muscle relaxants and neuromuscular blockade reversal agents.

Recomendations	ASA	ESAIC
Strongly recommended	Do not rely solely on clinical assessment of blockade reversal	Usage of muscle relaxants to facilitate tracheal intubation
	Choose quantitative monitoring over qualitative assessment for residual neuromuscular blockade	Usage of muscle relaxants to reduce pharyngeal and/or laryngeal injury following endotracheal intubation
	Avoid using ocular muscles for monitoring	Use a fast-acting muscle relaxant for RSII, such as succinylcholine 1 mg·kg^−1^ or rocuronium 0.9 to 1.2 mg·kg^−1^
	Use the adductor pollicis muscle for neuromuscular monitoring	Deep neuromuscular blockade if surgical conditions need to be improved
	Confirm a TOFR ≥ 0.9 before intubation when using quantitative monitoring	Use of ulnar nerve stimulation and quantitative NMM at the adductor pollicis muscle to exclude residual paralysis
	Consider using neostigmine as an alternative to sugammadex in cases of minimal depth of neuromuscular blockade	Use sugammadex to antagonize deep, moderate, and shallow neuromuscular blockade induced by aminosteroidal agents (rocuronium, vecuronium) (deep: post-tetanic count > 1 and TOF count = 0, moderate: TOF count = 1 to 3, shallow: TOF count = 4 and TOF ratio < 0.4)
		Advanced spontaneous recovery (i.e., TOF ratio > 0.2) before starting neostigmine-based reversal and to continue quantitative monitoring of neuromuscular blockade until a TOF ratio of more than 0.9 has been attained
Conditionally recommended (due to low strength of evidence)	In the use of atracurium or cisatracurium and minimal depth of neuromuscular blockade, consider using neostigmine to avoid residual blockade	
	In the absence of quantitative monitoring, after using neostigmine for blockade reversal, wait at least 10 min before extubation	

Notes and abbreviations: ASA—American Society of Anesthesiologists; ESAIC—European Society of Anaesthesiology and Intensive Care; RSII—rapid sequence induction intubation; TOF—train-of-four; TOFR—train-of-four ratio; NMM—neuromuscular monitoring. Recommendations adapted from: Peri-operative management of neuromuscular blockade: A guideline from the European Society of Anaesthesiology and Intensive Care [91] and American Society of Anesthesiologists Practice Guidelines for Monitoring and Antagonism of Neuromuscular Blockade: A Report by the American Society of Anesthesiologists Task Force on Neuromuscular Blockade [80]. Table has been taken from—[18] Radkowski P, Szewczyk M, Łęczycka A, Kowalczyk K, Kęska M, Stompór T. Impact of Liver Disease on Use of Muscle Relaxants in Anesthesia: A Comprehensive Review. Med Sci Monit. 2025;31:e945822. Published 2025 Jan 1. doi:10.12659/MSM.945822.

## Data Availability

No new data were created or analyzed in this study. Data sharing is not applicable to this article. All figures submitted have been created by the authors, who confirm that the images are original with no duplication and have not been previously published in whole or in part.
